# COVID-19: opening a new paradigm in thromboprophylaxis for critically ill patients?

**DOI:** 10.1186/s13054-020-03052-9

**Published:** 2020-06-11

**Authors:** Raquel Ferrandis, Juan V. Llau, Manuel Quintana, Pilar Sierra, Francisco Hidalgo, Concepción Cassinello, Aurelio Gómez-Luque

**Affiliations:** 1 Anaesthesiology and Critical Care Service, University Hospital La Fe, Valencia, Spain; 2 Anaesthesiology and Critical Care Service, University Hospital Doctor Peset, Valencia, Spain; 3 Intensive Medicine Service, University Hospital La Paz-Carlos III, Madrid, Spain; 4 Anaesthesiology and Critical Care Service, Fundació Puigvert, Barcelona, Spain; 5 Anaesthesiology and Critical Care Service, Clínica Universidad de Navarra, Pamplona, Spain; 6 Anaesthesiology and Critical Care Service, University Hospital Miguel Servet, Zaragoza, Spain; 7 Anaesthesiology and Critical Care Service, University Hospital Virgen de la Victoria, Málaga, Spain

To the Editor:

The novel infection caused by coronavirus SARS-CoV-2 determining COVID-19 disease causes alterations mainly in the respiratory system. Many reports have postulated a procoagulant state accompanying the respiratory distress with thrombosis at both venous and arterial levels [1]. The procoagulant pattern is characterized by hyperfibrinogenemia and elevated d-dimer levels, with mild thrombocytopenia and a moderately prolonged prothrombin time [2]. Although d-dimers are not specific indicators of clot formation, in combination with the other parameters, its elevation may suggest a systemic coagulation activation with an increase of thrombin generation and fibrinolysis.

A complex physiopathology has been proposed trying to explain this profile. Coming from the thromboinflammation concept, thrombin generation appears to be the key determinant of the thromboinflammatory response extent. The damaged endothelium, many blood cellular elements, and other activated hemostatic components are involved in this prothrombotic picture [3]. Microvascular thrombi impair the blood flow all over the body, with a vascular shunt due to capillary obstruction. This determines hypoxia and tissue dysfunction at several organs, being the lung the more affected one.

Many reports have highlighted the consequences of the pro-coagulant state, but evidence on how to prevent or even treat it is scarce. Prophylactic doses of low-molecular-weight heparin (LMWH) are recommended in most medical patients admitted to the hospital. Nevertheless, COVID-19 patients are probably out of these recommendations, and a new paradigm for the consideration of doses of LMWH could be open. Recent studies suggest the beneficial effect of the anticoagulation in severely ill COVID-19 patients, with an important reduction in mortality [4], opening the door to a new proposal increasing the dose of LMWH in this scenario (Fig. 1). Although the potential benefits of an increase of anticoagulation dose must be weighed and individualized, thromboprophylaxis management should consider the next proposals [5]:

1. All COVID-19 patients admitted to the hospital must be assessed on their thrombotic and hemorrhagic risk.
2. Unless contraindicated, LMWH at prophylactic dose must be administered.
3. When the pro-coagulant profile is confirmed, an extended or intermediate dose of LMWH should be considered, mainly in patients admitted to an ICU.
4. In the case of severe disease progression, with maintained high pro-coagulant parameters or high VTE suspicion, mainly if a certain diagnosis is not possible, the increase of the LMWH dose up to therapeutic one should be considered.
5. Therapeutic anticoagulation with LMWH should be the standard treatment when the diagnosis of any thrombotic event is confirmed.

**Fig. 1:**
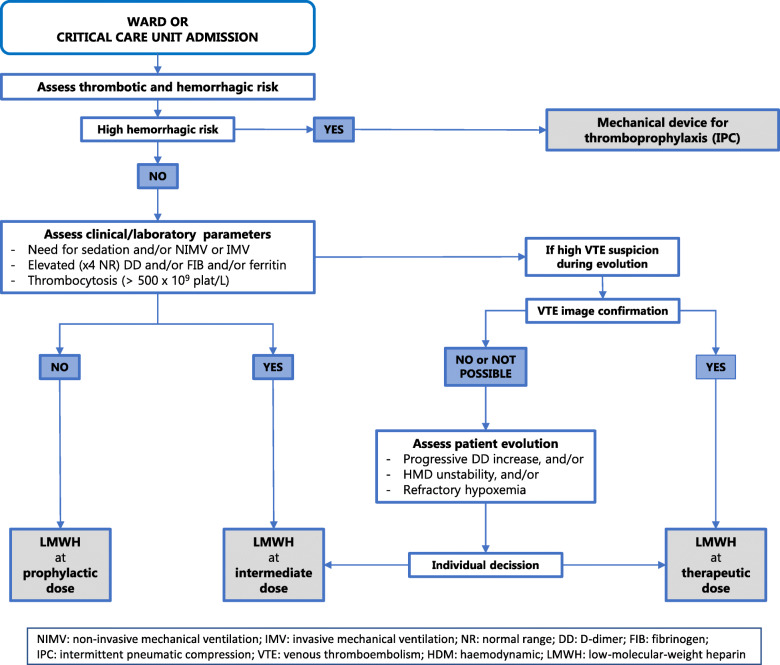
Summary proposal diagram for the management of thromboprophylaxis in critically ill COVID-19 patients

## Data Availability

Not applicable

## References

[1] Bikdeli B, Madhavan MV, Jimenez D, Chuich T, Dreyfus I, Driggin E, et al. COVID-19 and thrombotic or thromboembolic disease: implications for prevention, antithrombotic therapy, and follow-up. J Am Coll Cardiol. 2020. 10.1016/j.jacc.2020.04.031. 10.1016/j.jacc.2020.04.031 PMC7164881 32311448

[2] Guan W, Ni Z, Hu Y, et al. Clinical characteristics of coronavirus disease 2019 in China. N Engl J Med. 2020;382:1708–20. 10.1056/NEJMoa2002032 PMC7092819 32109013

[3] Jackson SP, Darbousset R, Schoenwaelder SM (2019). Thromboinflammation: challenges of therapeutically targeting coagulation and other host defense mechanisms. Blood..

[4] Paranjpe I, Fuster V, Lala A, Russak A, Glicksberg BS, Levin MA, et al. Association of treatment dose anticoagulation with in-hospital survival among hospitalized patients with COVID-19. J Am Coll Cardiol 2020 Epublished. 10.1016/j.jacc.2020.05.001. 10.1016/j.jacc.2020.05.001 PMC7202841 32387623

[5] Llau JV, Ferrandis R, Sierra P, Hidalgo F, Cassinello C, Gómez-Luque A, et al. SEDAR-SEMICYUC consensus recommendations on the management of haemostasis disturbances in severe patients with SARS-CoV-2 infection [article in Spanish]. Rev Esp Anestesiol Reanim. 2020; (in press). 10.1016/j.redar.2020.05.007.10.1016/j.redar.2020.05.007PMC724524232591185

